# Self-reported hypertension in Northern China: a cross-sectional study of a risk prediction model and age trends

**DOI:** 10.1186/s12913-018-3279-3

**Published:** 2018-06-19

**Authors:** Maolin Du, Shaohua Yin, Peiyu Wang, Xuemei Wang, Jing Wu, Mingming Xue, Huiqiu Zheng, Yajun Zhang, Danyan Liang, Ruiqi Wang, Dan Liu, Wei Shu, Xiaoqian Xu, Ruiqi Hao, Shiyuan Li

**Affiliations:** 1 School of Public Health, Inner Mongolia Medical University, Hohhot, 010110 China; 2 School of Public Health, Peking University, Beijing, 100191 China; 3 Department of nutrition and food hygiene, School of Public Health, Peking University, Beijing, 100191 China; 4 National Center for Chronic and Non-Communicable Disease Control and Prevention, Chinese Center for Disease Control and Prevention, Beijing, 100050 China; 5 School of Basic Medicine, Inner Mongolia Medical University, Hohhot, 010110 China; 6 College of Traditional Chinese Medicine, Inner Mongolia Medical University, Hohhot, 010110 China; 7 Beijing Tuberculosis and Thoracic Tumor Research Institute, Beijing Chest Hospital, Beijing, 101149 China

**Keywords:** Inner Mongolia, Self-reported, Hypertension, Risk factors, Risk score

## Abstract

**Background:**

Hypertension is a major risk factor for the global burden of disease, particularly in countries that are not economically developed. This study aimed to evaluate risk factors associated with self-reported hypertension among residents of Inner Mongolia using a cross-sectional study and to explore trends in the rate of self-reported hypertension.

**Methods:**

Multi-stage stratified cluster sampling was used to survey 13,554 participants aged more than 15 years residing in Inner Mongolia for the 2013 Fifth Health Service Survey. Hypertension was self-reported based on a past diagnosis of hypertension and current use of antihypertensive medication. Adjusted odds risks (*OR*s) of self-reported hypertension were derived for each independent risk factor including basic socio-demographic and clinical factors using multivariable logistic regression. An optimized risk score model was used to assess the risk and determine the predictive power of risk factors on self-reported hypertension among Inner Mongolia residents.

**Results:**

During study period, self-reported hypertension prevalence was 19.0% (2571/13,554). In multivariable analyses, both female and minority groups were estimated to be associated with increased risk of self-reported hypertension, adjusted *ORs* (95% *CI*) were 1.22 (1.08, 1.37) and 1.66 (1.29, 2.13) for other minority compared with Han, increased risk of self-reported hypertension prevalence was associated with age, marital status, drinking, BMI, and comorbidity. In the analyses calculated risk score by regression coefficients, old age (≥71) had a score of 12, which was highest among all examined factors. The predicted probability of self-reported hypertension was positively associated with risk score. Of 13,421 participants with complete data, 284 had a risk score greater than 20, which corresponded to a high estimated probability of self-reported hypertension (≥67%).

**Conclusions:**

Self-reported hypertension was largely related to multiple clinical and socio-demographic factors. An optimized risk score model can effectively predict self-reported hypertension. Understanding these factors and assessing the risk score model can help to identify the high-risk groups, especially in areas with multi-ethnic populations.

**Electronic supplementary material:**

The online version of this article (10.1186/s12913-018-3279-3) contains supplementary material, which is available to authorized users.

## Background

Hypertension (HTN) is a worldwide non-communicable disease epidemic and leads to further disabling comorbidities such as atherosclerosis, chronic kidney disease, and cardiac failure [1, 2]. The World Health Organization reported that HTN affected more than one billion people worldwide in 2010. Furthermore, HTN has led to the death of 9.4 million people and contributed to 7.0% of the global disability-adjusted life years, resulting in HTN being ranked third in terms of the global burden of disease [3]. Four large-scale population surveys have reported HTN rates in 1993, 1998, 2003 and 2008 were 5.11%, 7.73%, 13.58%, and 18.8%, respectively [4]. Although these previous findings indicate a lower prevalence in China than the global average, the total prevalence of HTN has increased over time. Many factors have been reported to be associated with HTN among residents of Inner Mongolia, including comorbidities, socioeconomic factors, and lifestyle characteristics, such as diabetes, age, drinking, body mass index (BMI, weight in kilograms divided by height in meters squared), and dietary habits [5–9]. However, the exact role and effect value of these factors in predicting HTN remain unclear.

Inner Mongolia is located in northern China, covering a large area and great diversity in terms of geographical climate, lifestyle, and demographic characteristics. Inner Mongolia is a multi- ethnic region, the majority population is Han Chinese. Han and minority groups have different cultural backgrounds, customs and lifestyle. Minority are more likely to eat more animal fat, milk products, drink strong wine, consume less fresh vegetables and fruits. Because the annual cold season is longer in this region than in other areas of China, Inner Mongolia is characterized by a lack of intake of fresh fruits and vegetables, a preference for pickled food, a high salt intake (> 20 g per day), and a high prevalence of smoking and drinking [10, 11]. In addition, because of a lack of local economic development, the level of culture and education are generally lower than other areas of the country, the level of medical care is poor, and residents lack awareness of health care, leading to poor public health conditions in terms of chronic diseases.

Studies from many countries have shown that lifestyle and public health interventions among different populations have consistent effects regarding the improvement of HTN [12], but there is no scoring system for assessing the risk associated with socio-demographic and health related factors. Therefore, a study exploring the self-reported HTN risk score among the residents of Inner Mongolia is required as part of the effort to reduce the HTN prevalence. The objective of this study was to assess the effect of a calculated risk score on self-reported HTN among the residents of multi-ethnic in Inner Mongolia.

## Methods

### Setting and target population

The present study was based on a population-based survey in Inner Mongolia, the Fifth Health Service Survey, which was conducted in 2013. This cross-sectional survey took place in a northern province of China (Inner Mongolia), and employed a self-reported questionnaire through a face-to-face household interview.

### Sampling procedure

Multi-stage stratified random cluster sampling was implemented in 12 cities in Inner Mongolia, including 56 counties, 80 sub-district offices, and 160 neighborhood committees. A total of 6090 households were enrolled in the study, a total of 15,387 Inner Mongolia residents completed the questionnaire, those who aged more than 15 years were 13,554 (88.09%).

### Questionnaire and measures

The survey was administered by trained health facility staff members, and the questionnaire comprised three sections. The first section contained questions about socio-demographic data, such as age, gender, ethnicity, occupation, and level of education. The second section contained questions regarding the health-related behaviors of the participants in the 12 months prior to the survey, including smoking, drinking, and BMI. The third section was disease status, including questions on self-reported HTN, diabetes, and other diseases.

For the purpose of this study, those living in poverty were defined as residents whose annual per capita income was below the threshold of the annual state per capita income (CNY 2300, which is equivalent to USD 362.82) or who lived in poor households. Level of education was categorized into three groups: low (junior high school or below), middle (high school or technical education), and high (tertiary education including specialist vocational training, undergraduate education, or postgraduate education). Marital status was specified as married, divorced/widowed, or single. Respondents were asked about their ethnicity (“Do you consider yourself Han/ Mongolian/ other?”). We then categorized ethnicity as Han, Mongolian, Hui, or other minority (e.g., Zhuang, Manchu, Bai).

Residents who had consumed alcohol (not included alcohol beverage) within the previous 12 months at the time of the interview were defined as drinkers, and those who had not consumed alcohol within this time period were defined as non-drinkers. Non-smokers, former smokers, and current smokers refer to those who reported never having smoked, those who previously smoked but quit, and those who currently consumed cigarettes, respectively. Residents also were asked about their depression condition (“Do you consider your self’s anxiety or depression condition is no/moderate/severe recently?”).

The outcome for this study was self-reported HTN. Hypertensive screening and diagnosis were firstly assessed by asking, “Have you ever been told by a doctor or health professional that you have HTN, also called high blood pressure?” Among those who answered yes to the above question, they were further asked, “Are you currently taking medicine for your HTN or HTN treatment?” HTN treatment refers to finding any antihypertensive medication while reviewing the medications the subjects was currently taking. Self-reported HTN was defined as having a past hypertensive diagnosis and a current HTN treatment.

### Statistical analysis

We used univariable logistic regression to identify the crude associations of socio-demographic characteristics and health-related behaviors with self-reported HTN among residents of Inner Mongolia. Multivariable logistic regression model was built using self-reported HTN as dependent variable (no self-reported HTN =0, self-reported HTN =1). The independent variables and their coding are shown in Table 1. Independent variables with *P*-values ≤0.10 in the univariable analysis were then included in the multivariable logistic regression model, performing in a backward manner using a significance level of *P*-values < 0.05. Estimating adjusted odds ratios (*ORs*) and 95% confidence interval (*CI*) using multivariable logistic regression with the following covariates: age, gender, ethnicity, level of education, occupation, marital status, smoking, drinking, BMI, and comorbidity. The selected variables in the final multivariable prediction model were considered as risk factors for self-reported HTN. Above analyses were performed using IBM SPSS 19.0. A 2-sided *P* value < 0.05 was considered significant.

**Table 1:** Independent variables and their coding

Variable	Categories
Gender	Male =0, Female =1
Poverty	Poor [annual per capita income below CNY 2300 (or USD 362.82) or lived in poor households] =1, Not-poor =0
Level of education	Low (junior high school and below) =1, Middle (high school and technical school) =2, High (senior for college, college, undergraduate and higher education) =3
Occupation	Unemployed =1, Retired =2, Employed =3
Ethnicity	Hui =1, Mongolian =2, Other minority (Zhuang, Manchu, Bai, etc.) =3, Han =4
Marital status	Single =1, Widowed/Divorced =2, Married =3
Smoking	Non-smoker (never having smoked previously) =1, Former smoker (previously smoked but quit) =2, Current smoker (currently consumed cigarettes) =3
Drinking	Yes = Drinker (consumed alcohol within the previous 12 months at the time of the interview) =1, No = Non-drinker (no consumed alcohol within this time period) =0
BMI (kg/m^2^)	BMI < 24 = 0, BMI ≥24 = 1
Comorbidity	No =0,Yes =1

STATA statistical software (version 13.0) was conducted to draw the nomogram. The nomogram was used to interpret the relation between probability of self-reported HTN and points, which is very easy and straightforward. We add points from point axis for each predictor to obtain a total point. And then project the total point to the axis of risk of self-reported HTN.

The area under the ROC curve (AUC) was used to estimate the predictive power of the final multivariable model, determine the cut-off value for the predicted probability of self-reported HTN, and calculate sensitivity and specificity of the predicted probability of self-reported HTN. The diagnostic accuracy of the multivariable model was considered non-significant when AUC was < 0.5, poor when AUC was 0.5–0.7, good when AUC was 0.7–0.9, and high when AUC was ≥0.9.

An optimized Framingham risk model was then used to calculate risk scores based on the selected risk factors and the corresponding coefficients from the multivariable prediction model. Using previously described methods for deriving risk scores, a points-based system was developed to estimate the probability of self-reported HTN (Additional file 1: Table S1) [13]. The following logistic regression model, obtained using backward stepwise model selection, was used to determine which variables to include in the risk score and the associated regression coefficients (*β*i):

$$ \mathrm{Logit}\ \Big(\mathrm{probability}\ \mathrm{of}\ \mathrm{Inner}\ \mathrm{Mongolia}\ \mathrm{resident}\ \mathrm{with}\ \mathrm{self}\hbox{-} \mathrm{reported}\ \mathrm{HTN}\mid {\mathrm{X}}_1,{\mathrm{X}}_2,{\mathrm{X}}_3,{\mathrm{X}}_4\dots {\mathrm{X}}_{\mathrm{n}} $$

For nominal variables, 0 and 1 were used as the reference values. When the upper or lower limit of the category range was not specified, the 1 percentile and 99 percentiles were used as the lower and upper limits for the midpoint calculations. A base risk profile (reference group, W_iREF_) was set to correspond to the lowest risk group for each variable. We then determined the distance between each reference value and the corresponding reference group in terms of regression units by multiplying the difference by the *β*_i_ coefficient (*β*_i_ [W_ij_ − W_iREF_]). To calculate the points corresponding to each of the risk factor categories, this value was divided by a constant (*B*). We used the constant value of X, which corresponded to the estimated risk associated with the risk factor that was the most influential predictor of self-reported HTN from the model (Additional file 1: Table S1). Risk scores were then categorized into three groups according to the estimated probability of self-reported HTN: low (< 16%), moderate (16–67%), and high (≥67%).

## Results

### Baseline characteristics of Inner Mongolia residents

The sample included 2571 (19.0%) residents with self-reported HTN in Inner Mongolia. The mean age of the residents was 47.17 years (standard deviation: 16.235 years, range: 15–96 years). The residents mainly concentrated in female (50.3%), Han (79.2%), employed (72.6%), and married (81.1%), and most had a low level of education (71.6%). Baseline characteristics varied across self-reported HTN status. As compared with residents without self-reported HTN, those who with self-reported HTN were more likely to be female, elder, minority, more likely to experience low education, widowed/divorced, more likely to be former smoker, more likely to have more BMI, and comorbidities (Tables 2 and 3).

**Table 2:** Baseline characteristics among Inner Mongolia residents with and without self-reported HTN

Characteristics	Study population (*n* = 13,554)	Self-reported HTN	No self-reported HTN
		(*n* = 2571)	(*n* = 10,983)
Age (X̄ ± S)	47.17 ± 16.24	59.65 ± 11.65	44.25 ± 15.76
Gender, n (%)			
Male	6721 (49.7)	1196 (46.6)	5525 (50.4)
Female	6814 (50.3)	1369 (53.4)	5445 (49.6)
Ethnicity, n (%)			
Hui	162 (1.2)	51 (2.0)	111 (1.0)
Mongolian	2115 (15.6)	342 (13.4)	1772 (16.2)
Other minority	539 (4.0)	112 (4.4)	427 (3.9)
Han	10,704 (79.2)	2059 (80.3)	8645 (78.9)
Level of education, n (%)			
Low	9695 (71.6)	2055 (80.0)	7640 (69.6)
Middle	1824 (13.5)	275 (10.7)	1549 (14.1)
High	2024 (14.9)	240 (9.3)	1784 (16.3)
Occupation, n (%)			
Unemployed	2070 (15.3)	557 (21.7)	1513 (13.8)
Retired	1634 (12.1)	659 (25.7)	975 (8.9)
Employed	9831 (72.6)	1352 (52.5)	8479 (77.3)
Marital status, n (%)			
Single	1562 (11.5)	41 (1.6)	1521 (13.9)
Widowed/Divorced	996 (7.4)	370 (14.4)	626 (5.7)
Married	10,982 (81.1)	2160 (84.0)	8822 (80.4)
Poverty, n (%)			
Poor	1366 (10.1)	340 (13.2)	1026 (9.3)
Not-poor	12,188 (89.9)	2231 (86.8)	9957 (90.7)
Smoking, n (%)			
Current smoking	3942 (32.3)	727 (31.3)	3215 (32.5)
Former smoking	483 (4.0)	144 (6.2)	339 (3.4)
No smoking	7780 (63.7)	1449 (62.5)	6331 (64.0)
Drinking, n (%)			
Yes	3247 (24.0)	583 (22.7)	2664 (24.3)
No	10,306 (76.0)	1988 (77.3)	8318 (75.7)
BMI, n (%)			
< 24	8250 (61.2)	1167 (45.6)	7083 (64.8)
≥ 24	5238 (38.8)	1391 (54.4)	3847 (35.2)
Comorbidity, n (%)			
Yes	1677 (12.4)	721 (28.0)	956 (8.7)
No	11,877 (87.6)	1850 (72.0)	10,027 (91.3)

**Table 3:** Univariable and multivariable analysis of predictors of self-reported HTN among residents of Inner Mongolia

Characteristic	Univariable Analysis *OR* (95%*CI*)	*P*	Multivariable Analysis *OR* (95%*CI*)^a^	*P*
Age	1.07 (1.07, 1.07)	< 0.001^*^	1.07 (1.07, 1.08)	< 0.001^*^
Gender (Male)				
Female	1.16 (1.07, 1.27)	0.001^*^	1.22 (1.08, 1.37)	< 0.001^*^
Ethnicity (Han)				
Hui	1.93 (1.38, 2.70)	< 0.001^*^	1.37 (0.79, 2.36)	0.267
Mongolian	0.81 (0.72, 0.92)	0.001^*^	1.15 (1.00, 1.33)	0.068
Other minority	1.10 (0.90, 1.36)	0.376	1.66 (1.29, 2.13)	< 0.001^*^
Level of education (High)				
Low	2.00 (1.73, 2.31)	< 0.001^*^		
Middle	1.32 (1.10, 1.59)	0.003^*^		
Occupation (Unemployed)				
Retired	2.31 (2.06, 2.59)	< 0.001^*^		
Employed	4.24 (3.78, 4.75)	< 0.001^*^		
Marital status (Single)				
Widowed, Divorced	21.93 (15.67, 30.68)	< 0.001^*^	1.82 (1.22, 2.72)	0.004^*^
Married	9.08 (6.64, 12.43)	< 0.001^*^	2.05 (1.42, 2.95)	< 0.001^*^
Poverty (No)				
Yes	1.48 (1.30, 1.69)	< 0.001^*^		
Smoking (No smoking)				
Current smoking	0.99 (0.90, 1.09)	0.811		
Former smoking	1.86 (1.52, 2.27)	< 0.001^*^		
Drinking (No)				
Yes	0.91 (0.83, 1.01)	0.091	1.19 (1.01, 1.32)	0.035^*^
BMI (< 24)				
≥ 24	2.20 (2.01, 2.39)	< 0.001^*^	2.49 (2.25, 2.76)	< 0.001^*^
Comorbidity (No)				
Yes	4.09 (3.67, 4.56)	< 0.001	2.06 (1.82, 2.34)	< 0.001^*^

Variables in brackets are reference categories

^*^P < 0.05

^a^Common confounders adjusted for in the multiple logistic regression included age, gender, ethnicity, level of education, occupation, marital status, smoking, drinking, BMI, and comorbidity

In the univariable analysis of the risk factors for self-reported HTN, we observed age, gender, ethnicity, level of education, occupation, marital status, poverty, smoking, drinking, BMI, and comorbidity were suggested to be statistically significant. Further multivariable modeling with these variables demonstrated that the *ORs* for self-reported HTN were 1.07 (95% *CI*: 1.07,1.08) for age (measured in years), 1.22 (95% *CI*: 1.08, 1.37) for female, 1.66 (95% *CI*: 1.29, 2.13) for minority (other minority vs. Han), 2.05 (95% *CI*: 1.42, 2.95) for married residents (married vs. single), 1.19 (95% *CI*: 1.01, 1.32) for drinkers, 2.49 (95% *CI*: 2.25, 2.76) for those with a high BMI (BMI ≥24), and 2.06 (95% *CI*: 1.82, 2.34) for those with comorbidities (Table 3). Thus, we derived the following multivariable logistic regression equation:

$$ {\displaystyle \begin{array}{l}\mathrm{Logit}\ \Big(\mathrm{probability}\ \mathrm{of}\ \mathrm{Inner}\ \mathrm{Mongolia}\ \mathrm{residents}\ \mathrm{with}\kern0.5em \mathrm{self}-\mathrm{reported}\ \mathrm{HTN}\mid {\mathrm{X}}_{\mathrm{Age}},{\mathrm{X}}_{\mathrm{Gender}},\\ {}{\mathrm{X}}_{\mathrm{Hui}},{\mathrm{X}}_{\mathrm{Mongolian}},{\mathrm{X}}_{\mathrm{Other}\ \mathrm{minority}},{\mathrm{X}}_{\mathrm{Married}},{\mathrm{X}}_{\mathrm{Other}\ \mathrm{marital}\ \mathrm{status}\left(\mathrm{widowed}\ \mathrm{or}\ \mathrm{divorced}\right)},{\mathrm{X}}_{\mathrm{drinker}},{\mathrm{X}}_{\mathrm{BMI}\ge 24},\\ {}{\mathrm{X}}_{\mathrm{With}\ \mathrm{comorbidity}}\Big)=-6.40+0.07\left(\mathrm{Age}\right)+0.20\left(\mathrm{Gender}\right)+0.31\left(\mathrm{Hui}\right)+0.14\left(\mathrm{Mongolian}\right)\\ {}+0.51\left(\mathrm{Other}\ \mathrm{minority}\right)+0.72\left(\mathrm{Married}\right)+0.60\left(\mathrm{Other}\ \mathrm{marital}\ \mathrm{status}\right)+0.17\left(\mathrm{Drinker}\right)\\ {}+0.91\left(\mathrm{BMI}\ge 24\right)+0.73\left(\mathrm{With}\ \mathrm{comorbidity}\right)\end{array}} $$

**Fig. 1:**
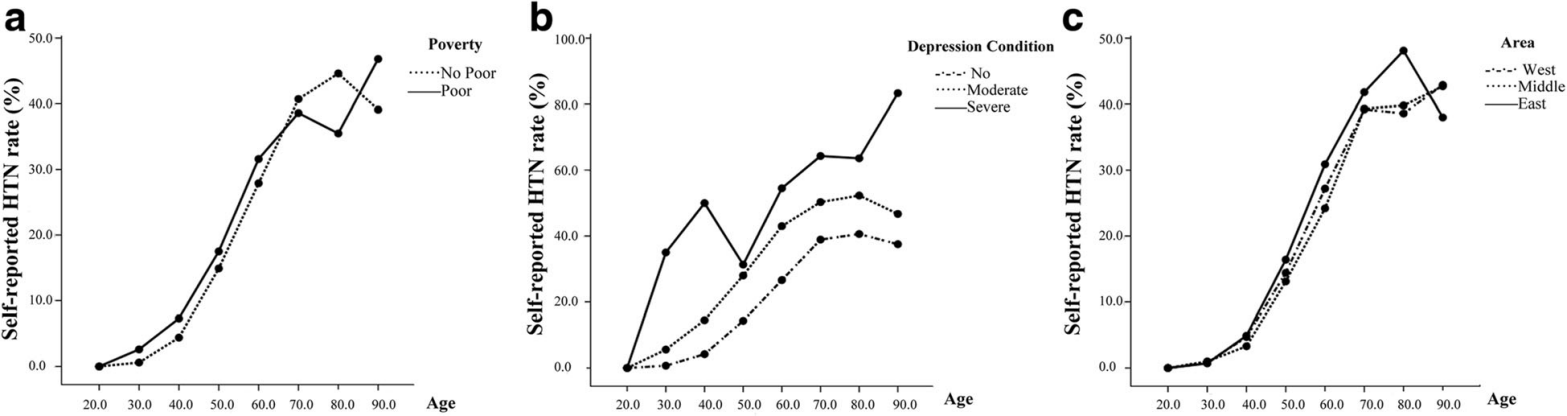
The trend of the self-reported HTN prevalence in Inner Mongolia, stratified by poverty status (**a**); The trend of the self-reported HTN prevalence in Inner Mongolia, stratified by depression condition (**b**); The trend of the self-reported HTN prevalence in Inner Mongolia, stratified by area (**c**)

Figure 1 suggests a relation of self-reported HTN with socio-demographic characteristics and age trend. The chi-square test for trend supported the hypothesis of differences in self-reported HTN prevalence over the age, stratified by poverty, depression, and area, which found the corresponding *P*-values were < 0.001 for poverty and depression but not for area. Rates of self-reported HTN were highest among poor residents. In the study, the rate of self-reported HTN increased linearly among poor and non-poor residents (*P-*values for trend < 0.001) (Fig. 1a). Likewise, the rate of self-reported HTN increased with age for residents in different depression conditions (*P* for trend < 0.001) (Fig. 1b). Variations in area did not result in a statistically significant differed self-reported HTN rate, but an increase trend of rate over age across the study areas could be seen (Fig. 1c).

### Risk factors associated with self-reported HTN among residents of Inner Mongolia

Total 11 variables with a *P* value ≤0.10 affected self-reported HTN in the univariable logistic regression were used to build a multivariable logistic regression model. The results suggested that old age, minority, widowed/divorced/married, and drinkers were more likely associated self-reported HTN. In addition, female, had comorbidities and high BMI (≥24) were the independent risk factors of self-reported HTN (Table 3). The sensitivity, specificity, and positive predictive value of the multivariable prediction model were 83.2%, 64.2%, and 35.2%, respectively. The multivariable prediction model suggested a higher predictive power for evaluating self-reported HTN, the AUC was 0.81 (95% *CI*: 0.80, 0.82), see Additional file 2: Figure S1.

### Risk score for self-reported HTN among Inner Mongolia residents with complete data

**Fig. 2:**
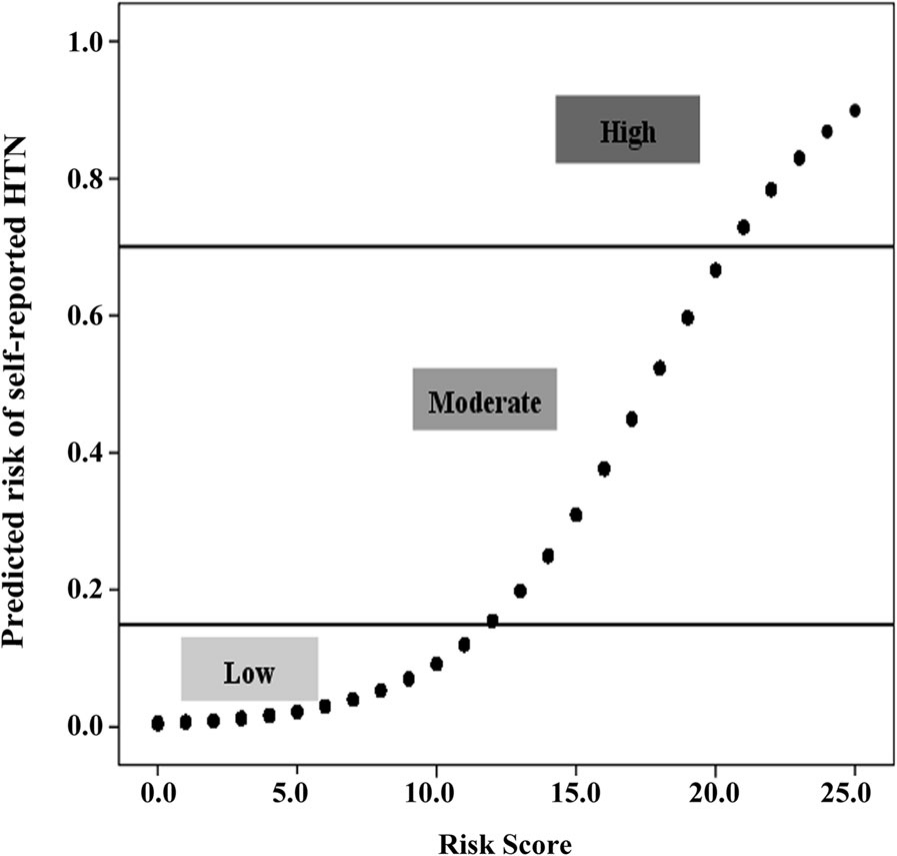
Risk of self-reported HTN and corresponding risk scores among residents of Inner Mongolia

**Fig. 3:**
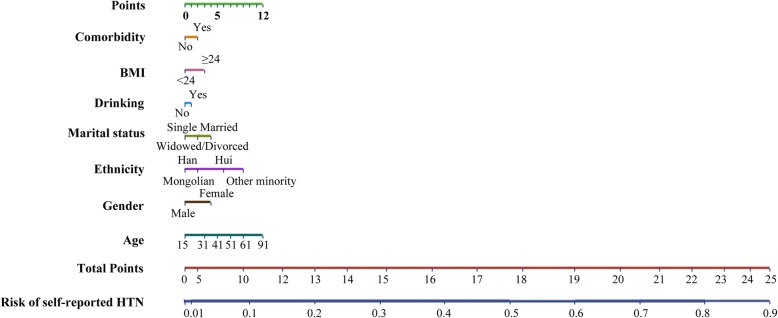
Nomogram model for self-reported HTN prediction in Inner Mongolia residents. (For example, a resident with age = 35, female, Han, married, BMI ≥ 24, with drinking and comorbidity, the total point is 14 read from the above nomogram, and the corresponding probability of self-reported HTN is 0.25)

Consequently, 13,421 residents (99%) who had complete data on risk factors of self-reported HTN were used to calculate risk scores. The risk factors between these residents and all participants did not differ substantially (Additional file 3: Table S2). The predicted probability of self-reported HTN was positively related to risk score (Fig. 2). And we observed aged ≥71 corresponded to a highest risk score of 12 points (Table 4). Of the 13,421 participants with complete data of predictors, 284 had a risk score greater than 20, which corresponded to an estimated probability of self-reported HTN of more than 67%. Although the highest possible score was 25, in this study, we categorized the highest scores as 21 or higher, which corresponded to a risk for 0.73 or above (greater than a high estimated probability of self-reported HTN of 67%). Finally, an estimated risk was calculated for each point total (Additional file 4: Table S3). Based on this seven-risk factors logistic regression model, we built the nomogram model for self-reported HTN prediction in Inner Mongolia residents. The nomogram model is showed in Fig. 3.

**Table 4:** Risk factors for self-reported HTN and corresponding risk scores estimated using multivariable logistic regression

Category level	Point value
Gender	
Male	0
Female	1
Age	
≤ 30	0
31–	3
41–	5
51–	7
61–	9
≥ 71	12
Ethnicity	
Han	0
Hui	1
Mongolian	1
Other minority	4
Marital status	
Single	0
Widowed/Divorced	2
Married	4
Drinking	
No	0
Yes	1
BMI (kg/m^2^)	
< 24	0
≥ 24	3
Comorbidity	
No	0
Yes	2

## Discussion

This study identified predictors significantly associated with self-reported HTN. Being female and minority groups were independent risk factors for self-reported HTN among residents of Inner Mongolia. The factor with the highest contribution to the risk score for self-reported HTN was being aged ≥71 years, which contributed 12 points to the risk score, on average. The risk prediction model showed a good predictive accuracy for self-reported HTN. This model is potential to have a positive effect on health services in Inner Mongolia, where both the economy and the healthcare conditions are relatively underdeveloped.

Several methods for assessing the risk of different diseases have previously been developed [14–16], but we found the optimized Framingham risk score model to be particularly valuable in our study. The Framingham Heart Profile (FHP) is currently applied worldwide in the assessment of various diseases [17], including coronary heart disease, metabolic syndrome, HTN, and tuberculosis [6, 18–20]. However, there are several limitations associated with directly applying the FHP [21] in Inner Mongolia, an autonomous region that is ecologically diverse and has residents of various minority groups with distinct regional and ethnic characteristics. Therefore, we used an optimized FHP to evaluate the risk of self-reported HTN among Inner Mongolia residents. The optimized FHP can serve as a readily available and useful tool that the majority of medical staff members and residents can use in the assessment of the risk of self-reported HTN and in identifying high-risk groups.

In this study, we suggested that age was a major risk factor for self-reported HTN among residents of Inner Mongolia. The risk of self-reported HTN increased by 0.07 (95% *CI*: 1.07, 1.08) unit for every one-year increase in age. Compared with residents aged < 30 years, the risk of self-reported HTN was higher among the other age groups. Of all of the risk factors examined, being aged ≥71 years was associated the highest addition to the risk score (12 points) for residents. This finding is consistent with reports from global epidemiological research, as well as results obtained in other parts of China [7, 22]. The result is mainly explained by factors such as arterial stiffness, thickness, loss of elasticity, and stenosis of the aorta and arterial wall among elders. In addition, older people are more likely to take less physically active and have more functional disorders [23, 24] that increase the risk of HTN.

Several studies have found that the risk of HTN is much higher among male than female [25, 26]. However, our study demonstrated that female was an independent risk factor for self-reported HTN among residents of Inner Mongolia. The risk score of female self-reported HTN was 1, corresponding to a higher risk (*OR*: 1.22, 95% *CI*: 1.08, 1.37). The difference in results between the present study and past findings may be related to differences in the sociocultural background of the study population, which lead to different psychological stresses. In China, people’s ideology is relatively conservative, in parallel, men and women are not equal in social or economic status. Although significant changes have accompanied economic development, many women still suffer from the greater stress. In such situations, the risks of developing physical illnesses and endocrine disorders because of various stresses are higher among women than men [27], which increase the risk of HTN or other chronic diseases. Our findings also revealed the distribution of risk factors of self-reported HTN and baseline characteristics on gender, which can help to identify that the female is an independent risk factor of self-reported HTN, which is not resulted due to self-reported bias. We found risk factors of self-reported HTN mainly concentrated in female, especially, proportion of female was higher than male in minority groups, female have a higher possibility in a low socioeconomic status and with comorbidity. These indicted the distribution of risk factors between self-reported HTN and female are consistent. The detailed information sees Additional file 5: Table S4.

Additionally, our study showed that married or widowed/divorced were independent risk factors for self-reported HTN, contributing 4 and 2 points to the risk score, respectively. This corresponded to a higher risk of self-reported HTN among residents of Inner Mongolia who were married (*OR*: 2.05, 95% *CI*: 1.42, 2.95) or widowed/divorced (*OR*: 1.82, 95% *CI*: 1.22, 2.72). The associations of culture, social and economic status, and marital status with the presence of chronic disease have been confirmed in several countries [28–30]. The findings of the present study suggested that female residents who married or widowed/divorced continue to experience serious work- and life-related stresses in Inner Mongolia.

A major finding of our study was that minority groups was an independent risk factor for self-reported HTN among residents of Inner Mongolia: The risk scores of Hui, Mongolian, and other minority status were 1, 1, and 4, respectively, corresponding to the *OR*s were 1.37 (95% *CI*: 0.79, 2.36), 1.15 (95% *CI*: 1.00, 1.33), and 1.66 (95% *CI*: 1.29, 2.13), respectively. Minority groups generally live in the eastern part of Inner Mongolia, which may explain the relatively higher rate of self-reported HTN in this region. In the United States, a 2005 survey conducted by the National Center for Chronic Disease Prevention and Health Promotion of the Centers for Disease Control and Prevention [31] showed that the age-adjusted self-reported HTN prevalence was 40.5% among non-Hispanic blacks and 27.4% among non-Hispanic whites. Our finding of ethnic disparity in self-reported HTN prevalence suggests that race may be an important risk factor. The Han and the minority populations in Inner Mongolia often have different lifestyles. Previous studies have demonstrated that dietary habits, genetics, and enduring lifestyle factors are associated with HTN [8, 32, 33]. Minority population often eats an unbalanced diet (e.g., excessive consumption of sodium, beef, mutton, and alcohol) [34–36]. The present study suggested that the risk score for self-reported HTN among drinkers in Inner Mongolia was 1, with a corresponding *OR* of 1.19 (95% *CI*: 1.01, 1.32). The long-term consumption of alcohol increases the risk of HTN by increasing the risk of arterial wall stiffness, which further lead to vascular atherosclerosis, cardiac disease, and cerebral vascular disease [37]. A study published in *The Lancet* indicated that low- and lower-/ middle-income countries contribute nearly two-thirds of the burden of chronic disease [38], which is consistent with our finding that the rate of self-reported HTN is higher among those living in poverty. Those living in poverty often have low levels of education and unhealthy lifestyles [39]. Future public health measures should be aimed at decreasing the prevalence of self-reported HTN among minority populations in Inner Mongolia by emphasizing prevention and improving control.

We also evaluated the association between BMI and self-reported HTN. Consistent with other studies, our study suggested that high BMI was an independent risk factor for self-reported HTN among residents of Inner Mongolia, with a risk score of 3 points and a corresponding *OR* of 2.49 (95% *CI*: 2.25, 2.77). In the Framingham Study, systolic blood pressure was found to increase 7 mmHg with a 10% increase in body weight in the majority of the population [40]. The JNC-7 report [41] recommends lifestyle improvements for all patients with prehypertension, including lose weight and increase exercise. We recommend focusing on high BMI as a target to implement proper interventions for the prevention and control of HTN.

Our study implied that comorbidity was also an independent risk factor for self-reported HTN among Inner Mongolia residents. Comorbidity had a self-reported HTN risk score of 2, with the corresponding *OR* of 2.06 (95% *CI*: 1.82, 2.34), compared with residents with no comorbidity. We also suggested increased risk of self-reported HTN was associated with more severe depression and old age, which was consistent with Jonas’s [42] finding that the incidence of HTN was higher among people with anxiety and depression who aged 45–64 years than low anxiety/depression scores. Bakris GL et al. found diabetes can increase incidence of HTN, and several antihypertensive agents are required to control blood pressure in patients with diabetes [43]. Routine screening for self-reported HTN among patients with comorbidities will likely help to earlier detection and treatment of HTN as well as improve clinical outcomes.

### Limitations

There are several limitations to this study. First, the study design was cross-sectional, the associations are not proof of causality, and reverse causality bias could be present. Additional cohort studies with follow-up data are necessary to strengthen the understanding of the associations identified here. Second, the majority of the information obtained was self-reported by the participants, which inevitably lead to reporting and recall bias although a standard questionnaire was used. Finally, we did not collect measures from laboratory tests, which may limit the clinical application of our findings.

## Conclusions

Despite the limitation, the study strengthens the understanding the relation of self-reported HTN with basic socio-demographic and clinical factors and among Inner Mongolia residents. Applying an optimized Framingham risk model could early screen the high-risk groups of self-reported HTN. Efforts to prognosis and prevent self-reported HTN in Inner Mongolia, especially multi-ethnic regions.

## Additional files

Additional file 1: **Table S1.** Risk score calculations for self-reported HTN in residents of Inner Mongolia. (DOC 51 kb)

Additional file 2: **Figure S1.** Receiver operating characteristic curves of final multivariable prediction model for residents with self-reported HTN. (TIF 63692 kb)

Additional file 3: **Table S2.** Differences between all the participants and those with complete data in all the selected predictors. (DOC 68 kb)

Additional file 4: **Table S3.** Estimated probability of self-reported HTN among residents of Inner Mongolia and corresponding risk score category. (DOC 56 kb)

Additional file 5: **Table S4.** Risk factors of self-reported HTN and baseline characteristics among male and female residents in Inner Mongolia. (DOC 44 kb)

## Abbreviations

95% CI: 95% Confidence interval
AUC: Area under the curve
BMI: Body mass index
FHP: Framingham Heart Profile
HTN: Hypertension
OR: Odds ratio
ROC: Receiver operating characteristics

## References

[1] Gaziano TA, Bitton A, Anand S, Abrahams-Gessel S, Murphy A (2010). Growing epidemic of coronary heart disease in low- and middle-income countries. Curr Probl Cardiol.

[2] Kearney PM, Whelton M, Reynolds K, Muntner P, Whelton PK, He J (2005). Global burden of hypertension: analysis of worldwide data. Lancet (London, England).

[3] Lim SS, Vos T, Flaxman AD, Danaei G, Shibuya K, Adair-Rohani H, Amann M, Anderson HR, Andrews KG, Aryee M (2012). A comparative risk assessment of burden of disease and injury attributable to 67 risk factors and risk factor clusters in 21 regions, 1990-2010: a systematic analysis for the global burden of disease study 2010. Lancet (London, England).

[4] guidelines Crcftpacoh. Chinese hypertension Guide Revision Commission. Chinese guidelines for the prevention and control of hypertension (revised in 2010). Chin Pract J Rural Doct. 2012;19(12):1–15.

[5] Yo K (2006). Moderate alcohol consumption does not prevent the hypertension among Korea: the 2001 Korean national health and nutrition examination survey. Korean J Community Nutr.

[6] Wang H, Fox CS, Troy LM, McKeown NM, Jacques PF (2015). Longitudinal association of dairy consumption with the changes in blood pressure and the risk of incident hypertension: the Framingham heart study. Br J Nutr.

[7] Mayega RW, Makumbi F, Rutebemberwa E, Peterson S, Ostenson CG, Tomson G, Guwatudde D (2012). Modifiable socio-behavioural factors associated with overweight and hypertension among persons aged 35 to 60 years in eastern Uganda. PLoS One.

[8] Elliott P, Kesteloot H, Appel LJ, Dyer AR, Ueshima H, Chan Q, Brown IJ, Zhao L, Stamler J (2008). Dietary phosphorus and blood pressure: international study of macro- and micro-nutrients and blood pressure. Hypertension.

[9] Dulskiene V, Kuciene R, Medzioniene J, Benetis R (2014). Association between obesity and high blood pressure among Lithuanian adolescents: a cross-sectional study. Ital J Pediatr.

[10] Zhou Y, Tian Y, Zhong C, Batu B, Tian X, Li H, Zhang M, Wang A, Zhang Y (2016). Combined effects of family history of CVD and heart rate on ischemic stroke incidence among inner Mongolians in China. Neurol Res.

[11] Huangfu X, Zhu Z, Zhong C, Bu X, Zhou Y, Tian Y, Batu B, Xu T, Wang A, Li H (2017). Smoking, hypertension, and their combined effect on ischemic stroke incidence: a prospective study among inner Mongolians in China. J Stroke Cerebrovasc Dis.

[12] Naing C, Aung K (2014). Prevalence and risk factors of hypertension in Myanmar: a systematic review and meta-analysis. Medicine.

[13] Sullivan LM, Massaro JM, D’Agostino RB (2004). Presentation of multivariate data for clinical use: the Framingham study risk score functions. Stat Med.

[14] Zlotnik A, Abraira V (2015). A general-purpose nomogram generator for predictive logistic regression models. Stata J.

[15] Zhou X, Qiao Q, Ji L, Ning F, Yang W, Weng J, Shan Z, Tian H, Ji Q, Lin L (2013). Nonlaboratory-based risk assessment algorithm for undiagnosed type 2 diabetes developed on a nation-wide diabetes survey. Diabetes Care.

[16] Lu CX, Chen HL, Shen WQ, Feng LP (2017). A new nomogram score for predicting surgery-related pressure ulcers in cardiovascular surgical patients. Int Wound J.

[17] D'Agostino RB, Wolf PA, Belanger AJ, Kannel WB (1994). Stroke risk profile: adjustment for antihypertensive medication. The Framingham Study. Stroke.

[18] Yousefzadeh G, Shokoohi M, Najafipour H, Shadkamfarokhi M (2015). Applying the Framingham risk score for prediction of metabolic syndrome: the Kerman coronary artery disease risk study, Iran. ARYA atherosclerosis.

[19] Jacob ST, Pavlinac PB, Nakiyingi L, Banura P, Baeten JM, Morgan K, Magaret A, Manabe Y, Reynolds SJ, Liles WC (2013). Mycobacterium tuberculosis bacteremia in a cohort of hiv-infected patients hospitalized with severe sepsis in Uganda-high frequency, low clinical suspicion [corrected] and derivation of a clinical prediction score. PLoS One.

[20] Joshi PH, Khokhar AA, Massaro JM, Lirette ST, Griswold ME, Martin SS, Blaha MJ, Kulkarni KR, Correa A, D'Agostino RB (2016). Remnant lipoprotein cholesterol and incident coronary heart disease: the Jackson heart and Framingham offspring cohort studies. J Am Heart Assoc.

[21] Jiuyi H, Yifeng C, Jiping G, Yangtai G, Yan W, Yongju Y, Xuehai Y, Fengying S (2013). Modified Framingham stroke profile in the prediction of the risk of stroke among Chinese. Chin J Cerebrovasc Dis.

[22] Joshi MD, Ayah R, Njau EK, Wanjiru R, Kayima JK, Njeru EK, Mutai KK (2014). Prevalence of hypertension and associated cardiovascular risk factors in an urban slum in Nairobi, Kenya: a population-based survey. BMC Public Health.

[23] Wakabayashi I (2012). Age-dependent influence of gender on the association between obesity and a cluster of cardiometabolic risk factors. Gend Med.

[24] Sa NN, Moura EC (2011). Overweight: socio-demographic and behavioral determinants in Brazilian adults, 2008. Cadernos de saude publica.

[25] Bozza R, Campos W, Barbosa Filho VC, Stabelini Neto A, Silva MP, Maziero RS (2016). High blood pressure in adolescents of Curitiba: prevalence and associated factors. Arq Bras Cardiol.

[26] Khanam MA, Lindeboom W, Razzaque A, Niessen L, Milton AH (2015). Prevalence and determinants of pre-hypertension and hypertension among the adults in rural Bangladesh: findings from a community-based study. BMC Public Health.

[27] Hu B, Liu X, Yin S, Fan H, Feng F, Yuan J (2015). Effects of psychological stress on hypertension in middle-aged Chinese: a cross-sectional study. PLoS One.

[28] Basu S, Millett C (2013). Social epidemiology of hypertension in middle-income countries: determinants of prevalence, diagnosis, treatment, and control in the WHO SAGE study. Hypertension.

[29] Fan AZ, Strasser SM, Zhang X, Fang J, Crawford CG (2015). State socioeconomic indicators and self-reported hypertension among US adults, 2011 behavioral risk factor surveillance system. Prev Chronic Dis.

[30] Ong KL, Cheung BM, Man YB, Lau CP, Lam KS (2007). Prevalence, awareness, treatment, and control of hypertension among United States adults 1999-2004. Hypertension.

[31] CDC Home. Racial/ethnic disparities in prevalence, treatment, and control of hypertension--United States, 1999–2002. Morb Mortal Wkly Rep. 2005;54(1):7–9. 15647724

[32] Johnson AG, Nguyen TV, Davis D (2001). Blood pressure is linked to salt intake and modulated by the angiotensinogen gene in normotensive and hypertensive elderly subjects. J Hypertens.

[33] McGuire HL, Svetkey LP, Harsha DW, Elmer PJ, Appel LJ, Ard JD (2004). Comprehensive lifestyle modification and blood pressure control: a review of the PREMIER trial. J Clin Hypertens (Greenwich).

[34] Chen L, Smith GD, Harbord RM, Lewis SJ (2008). Alcohol intake and blood pressure: a systematic review implementing a Mendelian randomization approach. PLoS Med.

[35] Sacks FM, Svetkey LP, Vollmer WM, Appel LJ, Bray GA, Harsha D, Obarzanek E, Conlin PR, Miller ER, Simons-Morton DG (2001). Effects on blood pressure of reduced dietary sodium and the dietary approaches to stop hypertension (DASH) diet. DASH-sodium collaborative research group. N Engl J Med.

[36] Schmieder RE, Messerli FH, Garavaglia GE, Nunez BD (1988). Dietary salt intake. A determinant of cardiac involvement in essential hypertension. Circulation.

[37] Yoon YS, Oh SW, Baik HW, Park HS, Kim WY (1969). Alcohol consumption and the metabolic syndrome in Korean adults: the 1998 Korean National Health and nutrition examination survey. Heriot-Watt University.

[38] Lozano R, Naghavi M, Foreman K, Lim S, Shibuya K, Aboyans V, Abraham J, Adair T, Aggarwal R, Ahn SY (2012). Global and regional mortality from 235 causes of death for 20 age groups in 1990 and 2010: a systematic analysis for the global burden of disease study 2010. Lancet.

[39] Prabhakaran D, Jeemon P, Reddy KS (2013). Commentary: poverty and cardiovascular disease in India: do we need more evidence for action?. Int J Epidemiol.

[40] Ashley FW, Kannel WB (1974). Relation of weight change to changes in atherogenic traits: the Framingham study. J Chronic Dis.

[41] Sood R, Sood AK (2005). The JNC 7 report on hypertension - critical analysis. Med Update.

[42] Jonas BS, Franks P, Ingram DD (1997). Are symptoms of anxiety and depression risk factors for hypertension? Longitudinal evidence from the National Health and nutrition examination survey I epidemiologic follow-up study. Arch Fam Med.

[43] Bakris GL (2001). A practical approach to achieving recommended blood pressure goals in diabetic patients. Arch Intern Med.

