# Supplementary material for: Self-reported hypertension in Northern China: a cross-sectional study of a risk prediction model and age trends
Source: BMC Health Serv Res. 2018 Jun 19;18:475. doi: 10.1186/s12913-018-3279-3 (PMC6006843; doi:10.1186/s12913-018-3279-3)
Supplement: Supplementary file 1 — Table S1. Risk score calculations for self-reported HTN in residents of Inner Mongolia. (DOC 51 kb) [file 12913_2018_3279_MOESM1_ESM.doc]

**Table S1 Risk score calculation for self-reported HTN in residents of Inner Mongolia calculations**

| **Predictor** | **Reference** | | **Referent** | | **|*β*_i_|** | **(W_ij_ – W_iREF_)** | | ***β*_i_** | | ***B*** | | **points** | |
| --- | --- | --- | --- | --- | --- | --- | --- | --- | --- | --- | --- | --- | --- |
|  | **(W_ij_)** | | **( W_iREF_)** | |  |  |  | **(W_ij_ – W_iREF_)** | |  |  | **[*β*_i_ (W_ij_ -W_iREF_)/*B*]** | |
| Gender |  |  | | 0.196 | |  | |  | | 0.340 | |  | |
| Male | 0 | Ref | |  | | 0 |  | |  | | 0 | |  |
| Female | 1 |  | |  | | 1 | 0.196 | |  | | 1 | |  |
| Age |  |  | | 0.068 | |  |  | |  | |  | |  |
| ≤30 | 22.5 | Ref | |  | |  |  | |  | | 0 | |  |
| 31~ | 35.5 |  | |  | | 13 | 0.884 | |  | | 3 | |  |
| 41~ | 45.5 |  | |  | | 23 | 1.564 | |  | | 5 | |  |
| 51~ | 55.5 |  | |  | | 33 | 2.244 | |  | | 7 | |  |
| 61~ | 65.5 |  | |  | | 43 | 2.924 | |  | | 9 | |  |
| ≥71 | 80.5 |  | |  | | 58 | 3.944 | |  | | 12 | |  |
| Ethnicity |  |  | |  | |  |  | |  | |  | |  |
| Han | 0 | Ref | |  | | 0 |  | |  | | 0 | |  |
| Hui | 1 |  | | 0.311 | | 1 | 0.311 | |  | | 1 | |  |
| Mongolian | 2 |  | | 0.135 | | 2 | 0.270 | |  | | 1 | |  |
| Other minority | 3 |  | | 0.505 | | 3 | 1.515 | |  | | 4 | |  |
| Marital status |  |  | |  | |  |  | |  | |  | |  |
| Single | 0 | Ref | |  | | 0 |  | |  | | 0 | |  |
| Widowed/  Divorced | 1 |  | | 0.598 | | 1 | 0.598 | |  | | 2 | |  |
| Married | 2 |  | | 0.716 | | 2 | 1.432 | |  | | 4 | |  |
| Drinking |  |  | |  | |  |  | |  | |  | |  |
| No | 0 | Ref | |  | | 0 |  | |  | | 0 | |  |
| Yes | 1 |  | | 0.173 | | 1 | 0.173 | |  | | 1 | |  |
| BMI (kg/m^2^) |  |  | |  | |  |  | |  | |  | |  |
| <24 | 0 | Ref | |  | | 0 |  | |  | | 0 | |  |
| ≥24 | 1 |  | | 0.913 | | 1 | 0.913 | |  | | 3 | |  |
| Comorbidity |  |  | |  | |  |  | |  | |  | |  |
| No | 0 | Ref | |  | | 0 |  | |  | | 0 | |  |
| Yes | 1 |  | | 0.725 | | 1 | 0.725 | |  | | 2 | |  |
| Intercept |  |  | | -6.401 | |  |  | |  | |  | |  |
