# Supplementary figures and images for: Self-reported hypertension in Northern China: a cross-sectional study of a risk prediction model and age trends
Source: BMC Health Serv Res. 2018 Jun 19;18:475. doi: 10.1186/s12913-018-3279-3 (PMC6006843; doi:10.1186/s12913-018-3279-3)

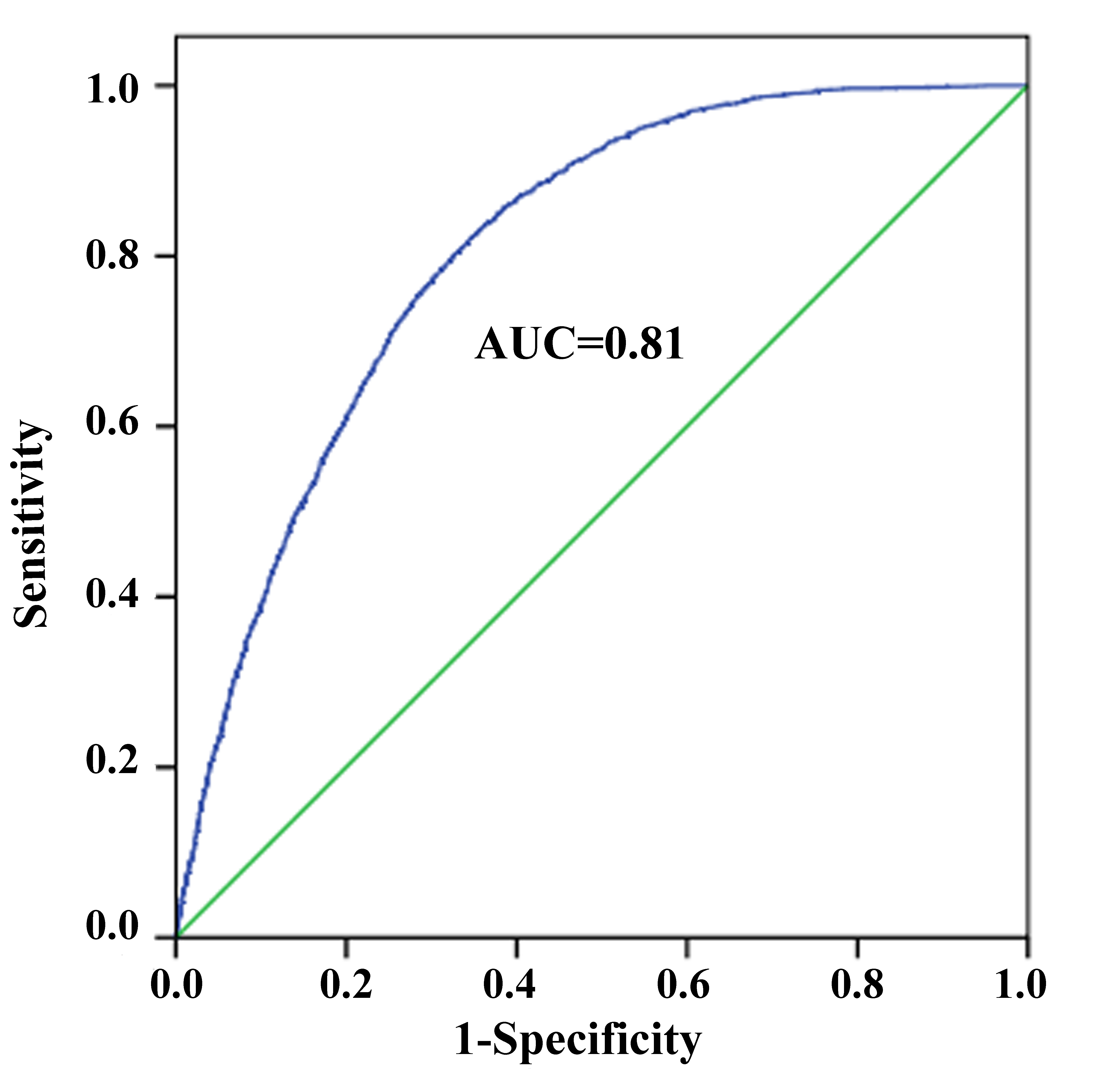

Supplement: Supplementary file 2 — Figure S1. Receiver operating characteristic curves of final multivariable prediction model for residents with self-reported HTN. (TIF 63692 kb) [file 12913_2018_3279_MOESM2_ESM.tif]
