# Supplementary material for: Self-reported hypertension in Northern China: a cross-sectional study of a risk prediction model and age trends
Source: BMC Health Serv Res. 2018 Jun 19;18:475. doi: 10.1186/s12913-018-3279-3 (PMC6006843; doi:10.1186/s12913-018-3279-3)
Supplement: Supplementary file 3 — Table S2. Differences between all the participants and those with complete data in all the selected predictors. (DOC 68 kb) [file 12913_2018_3279_MOESM3_ESM.doc]

**Table S2 Differences between all the participants and those with complete data in all the selected predictors**

| **Characteristics** | **Complete data** | | **Full data** | | **Statistics** | ***P*** |
| --- | --- | --- | --- | --- | --- | --- |
|  | **n(%)** | | **n(%)** | |  |  |
| Age (X±S)* | 47.20±16.213 | 47.17±16.235 | | -0.170 | | 0.865 |
| Gender |  |  | | 0.001 | | 0.975 |
| Male | 6667 (49.7) | 6721 (49.7) | |  | |  |
| Female | 6754 (50.3) | 6814 (50.3) | |  | |  |
| Ethnicity |  |  | | 0.009 | | 1.000 |
| Han | 10625 (79.2) | 10704 (79.2) | |  | |  |
| Hui | 162 (1.2) | 162 (1.2) | |  | |  |
| Mongolian | 2101 (15.7) | 2115 (15.6) | |  | |  |
| Other minority | 533 (4.0) | 539 (4.0) | |  | |  |
| Occupation |  |  | | 0.001 | | 0.999 |
| Unemployed | 2049 (15.3) | 2070 (15.3) | |  | |  |
| Retired | 1619 (12.1) | 1634 (12.1) | |  | |  |
| Employed | 9744 (72.7) | 9831 (72.6) | |  | |  |
| Marital status |  |  | | 0.031 | | 0.985 |
| Single | 1547 (11.5) | 1562 (11.5) | |  | |  |
| Widowed, Divorced | 980 (7.3) | 996 (7.4) | |  | |  |
| Married | 10894 (81.2) | 10982 (81.1) | |  | |  |
| alcohol use |  |  | | 0.012 | | 0.913 |
| Yes | 3223 (24.0) | 3247 (24.0) | |  | |  |
| No | 10198 (76.0) | 10306 (76.0) | |  | |  |
| BMI |  |  | | 0.004 | | 0.950 |
| <24 | 8204 (61.1) | 8250 (61.2) | |  | |  |
| ≥24 | 5217 (38.9) | 5238 (38.8) | |  | |  |
| Comorbidities |  |  | | 0.001 | | 0.978 |
| Yes | 1662 (12.4) | 1677 (12.4) | |  | |  |
| No | 11759 (87.6) | 11877 (87.6) | |  | |  |
| Hypertension |  |  | | <0.001 | | 0.997 |
| Yes | 2546 (19.0) | 2571 (19.0) | |  | |  |
| No | 10875 (81.0) | 10983 (81.0) | |  | |  |

Note:* t-test
