# Supplementary material for: Self-reported hypertension in Northern China: a cross-sectional study of a risk prediction model and age trends
Source: BMC Health Serv Res. 2018 Jun 19;18:475. doi: 10.1186/s12913-018-3279-3 (PMC6006843; doi:10.1186/s12913-018-3279-3)
Supplement: Supplementary file 4 — Table S3. Estimated probability of self-reported HTN among residents of Inner Mongolia and corresponding risk score category. (DOC 56 kb) [file 12913_2018_3279_MOESM4_ESM.doc]

**Table S3 Estimated probability of self-reported HTN among residents of Inner Mongolia and corresponding risk score category**

| **Risk score** | **Estimated probability of self-reported HTN** | | | **Risk score category** | | | **Number of subjects** | |
| --- | --- | --- | --- | --- | --- | --- | --- | --- |
| 0 | | <0.01 | | | 1 (low) | | 320 | |
| 1 | | <0.01 | | | 1 (low) | | 501 | |
| 2 | | <0.01 | | | 1 (low) | | 144 | |
| 3 | | | 0.01 | | | 1 (low) | | 134 |
| 4 | | | 0.02 | | | 1 (low) | | 261 |
| 5 | | | 0.02 | | | 1 (low) | | 525 |
| 6 | | | 0.03 | | | 1 (low) | | 192 |
| 7 | | | 0.04 | | | 1 (low) | | 306 |
| 8 | | | 0.05 | | | 1 (low) | | 1017 |
| 9 | | | 0.07 | | | 1 (low) | | 645 |
| 10 | | | 0.09 | | | 1 (low) | | 1264 |
| 11 | | | 0.12 | | | 1 (low) | | 1112 |
| 12 | | | 0.16 | | | 2 (moderate) | | 1329 |
| 13 | | | 0.20 | | | 2 (moderate) | | 1250 |
| 14 | | | 0.25 | | | 2 (moderate) | | 1113 |
| 15 | | | 0.31 | | | 2 (moderate) | | 1024 |
| 16 | | | 0.38 | | | 2 (moderate) | | 692 |
| 17 | | | 0.45 | | | 2 (moderate) | | 758 |
| 18 | | | 0.52 | | | 2 (moderate) | | 256 |
| 19 | | | 0.60 | | | 2 (moderate) | | 294 |
| 20 | | | 0.67 | | | 3 (high) | | 151 |
| 21+ | | | 0.73 | | | 3 (high) | | 133 |
