# Supplementary material for: Self-reported hypertension in Northern China: a cross-sectional study of a risk prediction model and age trends
Source: BMC Health Serv Res. 2018 Jun 19;18:475. doi: 10.1186/s12913-018-3279-3 (PMC6006843; doi:10.1186/s12913-018-3279-3)
Supplement: Supplementary file 5 — Table S4. Risk factors of self-reported HTN and baseline characteristics among male and female residents in Inner Mongolia. (DOC 44 kb) [file 12913_2018_3279_MOESM5_ESM.doc]

**Table S4 Risk factors of self-reported HTN and baseline characteristics among male and female residents in Inner Mongolia**

| Variable |  | Male n (%) |  | Female n (%) |  | Statistics |  | *P* |
| --- | --- | --- | --- | --- | --- | --- | --- | --- |
| Age |  | 47.08±16.36 |  | 47.25±16.11 |  | -0.600 |  | 0.549 |
| BMI |  | 23.56±3.52 |  | 23.12±3.47 |  | 7.312 |  | <0.001** |
| Poverty, n (%) |  |  |  |  |  | 0.382 |  | 0.537 |
| Poor |  | 666 (48.9) |  | 697 (51.1) |  |  |  |  |
| Non-poor |  | 6055 (49.7) |  | 6117 (50.3) |  |  |  |  |
| Ethnicity, n (%) |  |  |  |  |  | 9.753 |  | 0.021* |
| Hui |  | 68 (42.0) |  | 94 (58.0) |  |  |  |  |
| Mongolian |  | 1022 (48.3) |  | 1093 (51.7) |  |  |  |  |
| Other minority |  | 247 (45.9) |  | 291 (54.1) |  |  |  |  |
| Han |  | 5368 (50.2) |  | 5319 (49.8) |  |  |  |  |
| Level of education, n (%) |  |  |  |  |  | 31.859 |  | <0.001** |
| Low |  | 4681 (48.3) |  | 5008 (51.7) |  |  |  |  |
| Middle |  | 1004 (55.3) |  | 812 (44.7) |  |  |  |  |
| High |  | 1033 (51.2) |  | 986 (48.8) |  |  |  |  |
| Occupation, n (%) |  |  |  |  |  | 278.27 |  | <0.001** |
| Unemployed |  | 691 (33.4) |  | 1378 (66.6) |  |  |  |  |
| Retired |  | 775 (47.6) |  | 853 (52.4) |  |  |  |  |
| Employed |  | 5249 (53.5) |  | 4570 (46.5) |  |  |  |  |
| Marital status, n (%) |  |  |  |  |  | 148.208 |  | <0.001** |
| Single |  | 925 (59.3) |  | 635 (40.7) |  |  |  |  |
| Widowed/Divorced |  | 343 (34.6) |  | 649 (65.4) |  |  |  |  |
| Married |  | 5448 (49.7) |  | 5521 (50.3) |  |  |  |  |
| Drinking, n (%) |  |  |  |  |  | 2769.08 |  | <0.001** |
| Yes |  | 2917 (89.9) |  | 326 (10.1) |  |  |  |  |
| No |  | 3804 (37.0) |  | 6487 (63.0) |  |  |  |  |
| Comorbidity, n (%) |  |  |  |  |  | 20.038 |  | <0.001** |
| Yes |  | 746 (44.5) |  | 929 (55.5) |  |  |  |  |
| No |  | 5975 (50.4) |  | 5885 (49.6) |  |  |  |  |

**P*<0.05 ***P*<0.001 (t test for continuous variables and chi-square test for categorical variables).
